# Lorazepam-induced full resolution of catatonia and psychosis in Parkinson’s disease with acromegaly: a case report

**DOI:** 10.3389/fpsyt.2026.1806346

**Published:** 2026-05-04

**Authors:** Aasal Alnafisi, Ghaida Alharbi, Rami Ahmad, Suhaib Radi, Madihah Alhubayshi, Ahmed AlGhamdi

**Affiliations:** 1 College of Medicine, King Saud Bin Abdulaziz University for Health Sciences, Jeddah, Saudi Arabia; 2 King Abdullah International Medical Research Center, Jeddah, Saudi Arabia; 3 Mental Health Section, Department of Medicine, King Abdulaziz Medical City, Ministry of National Guard-Health Affairs, Jeddah, Saudi Arabia; 4 Department of Internal Medicine, Division of Endocrinology, King Abdulaziz Medical City, Ministry of the National Guard-Health Affairs, Jeddah, Saudi Arabia; 5 Department of Neuroscience, King Abdulaziz Medical City, Ministry of the National Guard-Health Affairs, Jeddah, Saudi Arabia; 6 Department of Radiology, King Abdulaziz Medical City, Ministry of the National Guard-Health Affairs, Jeddah, Saudi Arabia

**Keywords:** acromegaly, catatonia, lorazepam, Parkinson’s disease, psychosis

## Abstract

**Background:**

Parkinson’s disease (PD) is a neurodegenerative disorder characterized by the loss of dopamine-producing neurons, which results in motor issues such as tremors, stiffness, and slowness of movement. In addition to experiencing non-motor symptoms like psychosis. Catatonia, a psychomotor syndrome that is resulted from dopamine and cerebral cortical dysfunction, is considered a rare manifestation of PD patients. Acromegaly, a hormonal disorder caused by excessive growth hormone, often due to pituitary adenomas, can worsen cognitive and psychiatric symptoms. This case report demonstrates how lorazepam resulted in the full resolution of both catatonia and psychosis in a PD patient with acromegaly.

**Case presentation:**

A 78-year-old woman, known case of PD and major depressive disorder, presented to our hospital after the discontinuation of all her medications and worsening of her symptoms. She had significant neuropsychiatric symptoms that were resistant to treatment with various approaches. Her symptoms, which were consistent with catatonia and psychosis, was completely alleviated by taking lorazepam, along with restarting levodopa. Brain Magnetic Resonance Imaging (MRI) showed a pituitary macroadenoma, and hormonal studies verified acromegaly. Within six days of initiating lorazepam treatment, both catatonia and psychosis completely resolved, with an overall improvement of approximately 50%.

**Conclusion:**

This case highlights the importance of recognizing catatonia and psychosis as possible complications of PD, especially in the context of medication withdrawal and comorbid acromegaly. These neuropsychiatric symptoms were successfully resolved by lorazepam, demonstrating the medication’s effectiveness in treating complex co-occurring disorders.

## Introduction

Parkinson’s disease (PD) is a neurodegenerative disorder characterized by the degeneration and loss of the dopaminergic neurons in the *substantia nigra pars compacta*, resulting in dopamine deficiency that contributes to the emergence of hallmark motor symptoms such as tremor, bradykinesia, rigidity, and postural instability (1, 2) Non-motor symptoms, encompassing neuropsychiatric manifestations, are associated with the involvement of other neurotransmitter systems, which are serotonergic, noradrenergic, and cholinergic pathways (3). Psychotic symptoms impact approximately 70% of PD individuals after 20 years, due to disease progression or dopaminergic therapy (4). Catatonia is a psychomotor syndrome linked to numerous medical, neurological, and psychiatric conditions; however, it is rarely seen in PD (5, 6). The motor dysfunction in PD arises from the disruption of “bottom-up” dopaminergic pathway, where dopamine deficiency in the ganglia (a subcortical region) impairs signals moving up to the motor cortex of the brain, therefore hindering movement control. In contrast, catatonia, which represents “top-down” cortical dysregulation, where deficits in the cortex, particularly within the orbitofrontal region, compromise inhibitory control over subcortical structures (7). Acromegaly, most commonly caused by a pituitary adenoma, leads to excess growth hormone and increased IGF-1 levels, contributing to creating systemic symptoms and cognitive impairments (8–10). While there are an extensive research on PD-related psychosis, the relationship between PD and acromegaly in neuropsychiatric presentations remains inadequately comprehended. This case illustrates the successful use of lorazepam in treating catatonia and psychosis in a patient with PD and acromegaly.

## Case presentation

### History of presenting illness

This is a case of 78-year-old woman from an urban area, presented to a tertiary hospital’s Emergency Department (ED) with acute confusion, disorientation, and agitation. Her symptoms began two days before presentation and were preceded by the abrupt discontinuation of all her medications two weeks prior, which her family attributed to a gradual deterioration in her mental state and lack of response.

### Past medical history

Her medical history includes Parkinson’s disease (diagnosed a year ago, though symptoms began 10 years ago with right-hand and buccal tremors), major depressive disorder (diagnosed 8 months ago), left eye blindness due to glaucoma, diabetes mellitus, hypertension, and a recent pulmonary embolism PE) treated 2 months prior. For Parkinson’s disease, she was prescribed levodopa/carbidopa/entacapone and amantadine. She was then given escitalopram to help her with depression, and it resulted in clinical improvement. However, her family stopped giving it to her after 6 weeks because they were worried she might get addicted to it. Her symptoms deteriorated and came back with psychosis, necessitating restarting escitalopram and adding sulpiride. After she was admitted to her PE admission, sulpiride was switched to quetiapine by her psychiatrist. She remained clinically stable, though not fully resolved, until she discontinued all medications 2 weeks before her ED visit, after which she became increasingly agitated, disoriented, and had poor appetite and sleep.

### Family history

Her family history is significant for psychiatric conditions: One daughter is being treated for major depressive disorder with escitalopram, and her son (the patient’s grandson) has panic disorder.

### Social history

She is married and lives with her husband and youngest son. she has eight daughters and two sons. Her relationship with her husband was described as long-standing, strained relationship; when upset or when he refused her requests, she would hit her head against the wall repeatedly.

### Clinical course

**Figure 1:**
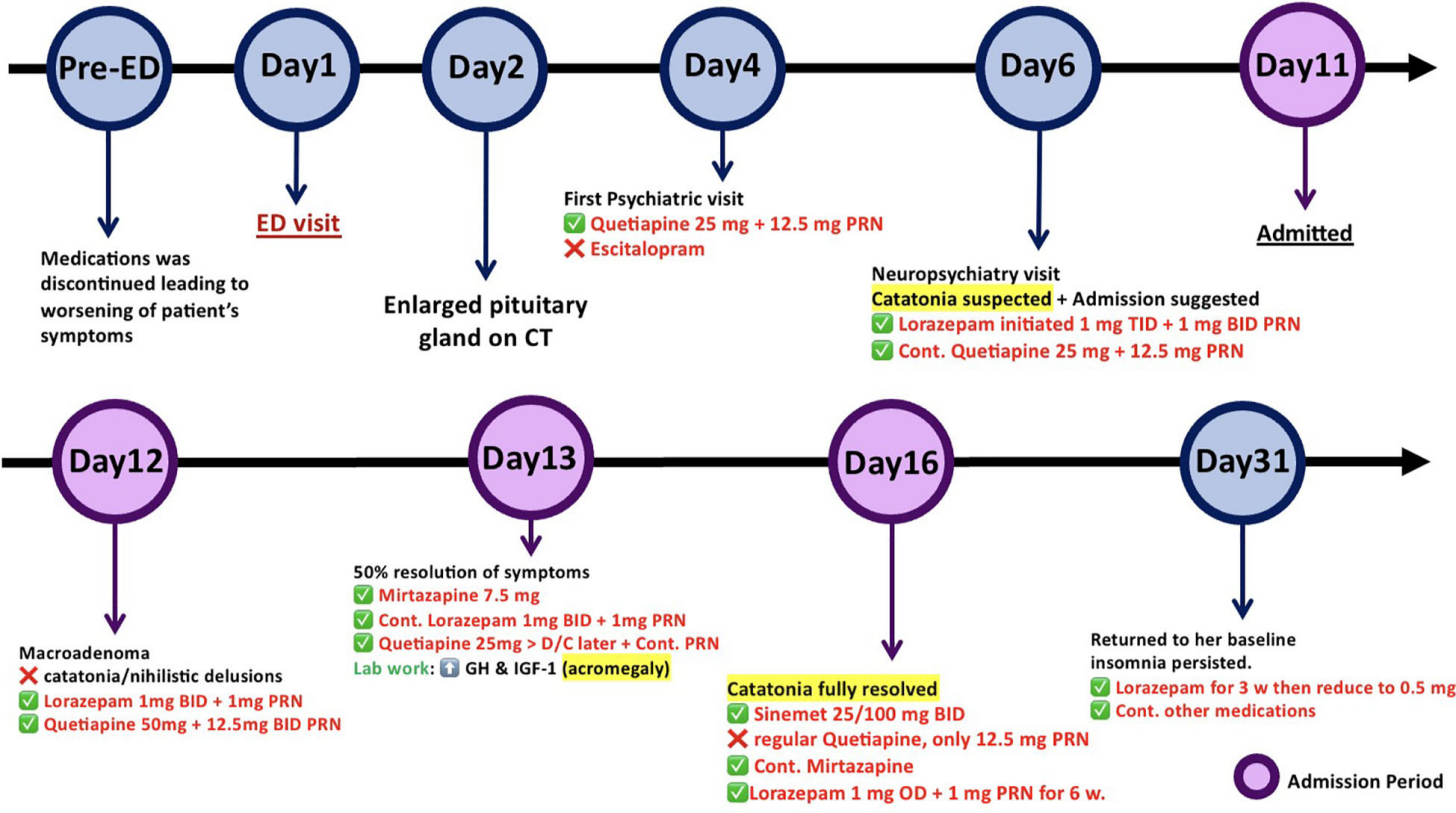
Timeline illustrating the most significant events and interventions of the case.

To provide a clear overview of her clinical journey, a timeline summarizing key events and interventions from her initial ED presentation to the resolution of her symptoms is shown in **Figure 1**. At the ED, she was confused, disoriented, with inappropriate speech, and agitated. There was nothing remarkable about her physical examination, and her Glasgow Coma Scale (GCS) score was 14 out of 15. Initial investigations revealed no acute abnormalities, and she returned to her baseline consciousness with basic supportive care. She was discharged with referrals to the outpatient care of neurology and psychiatry. Nevertheless, an unexpected finding on a brain CT revealed an enlarged pituitary gland, prompting additional assessment by a pituitary MRI and hormonal profile. There were also referrals to endocrinology and neurosurgery. On Day 4 after discharge, her son and daughter attended her psychiatric appointment on her behalf. They reported that she had been experiencing excessive daytime sleepiness while taking quetiapine 25 mg nightly and 12.5 mg as needed during the day. They claimed that she had been disoriented, irritable, shouting, excessively talkative, and only recognizing her son and daughter. They observed that she had been forgetful over the past 7 months.

Escitalopram was discontinued at this point, and a neuropsychiatric clinic appointment was arranged.

**Figure 2:**
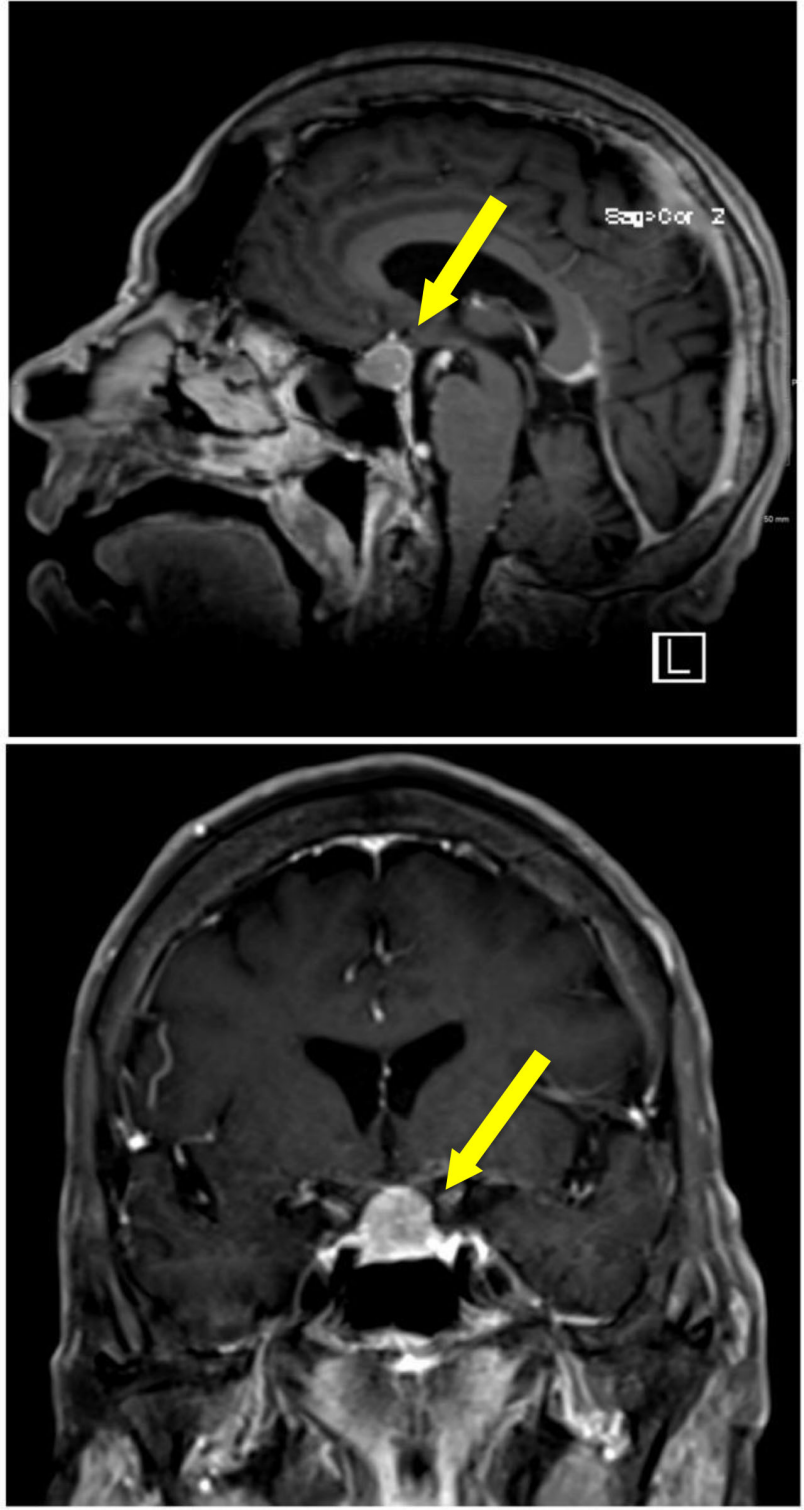
Sagittal and coronal MRI of the pituitary demonstrate homogeneously enhancing enlarged pituitary gland - pituitary macroadenoma.

On Day 6, in a follow-up virtual consultation, her son reported that her symptoms had deteriorated. These symptoms included increased physical aggression, disorganized behavior, nihilistic delusions (for example, the belief she was dead), echolalia, rigidity, posturing, mannerisms, and fluctuating behavior. The presence of catatonia was suspected. Key differentials, including neuroleptic malignant syndrome, dopamine agonist withdrawal syndrome, and worsening Parkinsonian symptoms following medication withdrawal, were carefully considered. However, the absence of fever, marked autonomic instability, and severe rigidity, along with the prompt clinical response to lorazepam, favored catatonia. She had no history of dementia; however, she experienced episodes of *delirium*, which were likely related to the withdrawal of her Parkinson’s medications. Lorazepam was initiated orally at a dose of 1 mg three times daily, with an additional 1 mg dose given twice daily as needed, along with quetiapine. It was started 6 days before her scheduled admission. On Day 11, she was admitted after showing a marked clinical improvement. During the admission, a psychiatry consultation confirmed full resolution of catatonia and psychosis within 6 days of lorazepam initiation—representing approximately a 50% overall improvement. However, she continued to experience sleep difficulties and death wishes without suicidal intent. Disorientation to place persisted as well. MRI of the pituitary revealed a homogeneously enhancing sellar and suprasellar mass (1.4 x 2.2 x 1.5 cm) displacing the optic chiasm, suggestive of a pituitary macroadenoma (**Figure 2**). Additional findings included ventricular and subcortical angiopathy and old bilateral midbrain infarctions. These results led to hormonal testing and neurosurgery consultations. Acromegaly was confirmed by the pituitary hormonal panel, which showed elevated growth hormone (GH) at 29.2 ng/mL (normal < 5 ng/mL) and insulin-like growth factor 1 (IGF-1) at 377.9 ng/mL (normal = 19-210 ng/mL). In addition, the patient demonstrated characteristic features, including growth of the jaw and hands. Consequently, treatment with lanreotide 120 mg was commenced as a monthly subcutaneous injection. Following a neurosurgical consultation, her family preferred conservative care, with a follow-up MRI scheduled in 3 months. The treatment plan was modified on Day 12. The lorazepam dosage was sustained at 1 mg twice daily, with an additional 1 mg provided as needed for breakthrough symptoms. The dosage of quetiapine was elevated to 50 mg at night, with 12.5 mg given twice daily if required for agitation. A follow-up on Day 13 revealed that the catatonia, agitation, and psychosis had all fully subsided, indicating a sustained improvement. The residual symptoms included mild irritability, forgetfulness, insomnia, and weakness. Due to quetiapine’s lack of effectiveness, it was gradually decreased, while mirtazapine 7.5 mg was started for mood and sleeplessness. Also, lorazepam 1 mg twice daily was continued for the relapse prevention. By Day 16, the patient was deemed fit for discharge. She was oriented, slept well, was eating normally, and showed no signs of catatonia or agitation. Quetiapine was discontinued except for use as needed. Lorazepam was reduced to 1 mg at bedtime with additional doses as needed, to be continued for at least 6 weeks. Furthermore, mirtazapine was continued. The neurology team resumed the administration of levodopa/carbidopa 25/100 mg twice daily, and no adverse effects were observed. Diabetes management was reviewed during an endocrinology follow-up on day 20. Her HbA1c was 9.0%, and she was on insulin degludec and aspart. Hormonal studies were within normal postmenopausal ranges. However, in the context of a macroadenoma, subtle pituitary dysfunction cannot be excluded. By day 31, in neuropsychiatric clinic follow-up, the patient had returned to her baseline state. She remained on levodopa/carbidopa, lorazepam, and mirtazapine, reporting stable mood and light sleep without daytime fatigue. Cognitive assessment using the Rowland Universal Dementia Assessment Scale (RUDAS) (11), scored 22/30. The patient’s domain scores were as follows: memory (recall) 2/8 visuospatial orientation 5/5, praxis 1/2, visuoconstructional drawing 2/3, judgment 4/4, and language (verbal fluency) 8/8. Behavioral activation and cognitive rehabilitation were encouraged. Hormonal levels had normalized. Lorazepam tapering was planned over the next 3 weeks.

## Discussion

This case illustrates a diagnostically challenging and rare presentation of catatonia and psychosis in a patient with coexisting Parkinson’s disease (PD) and acromegaly. To our knowledge, this is the first case to document the successful use of lorazepam in managing catatonia and psychosis in the context of acromegaly and Parkinson’s disease. A focused literature search using databases such as PubMed and Google Scholar revealed no similar reports. However, we acknowledge the potential limitations of such searches, including the existence of unpublished or non-indexed cases. The case emphasizes the importance of recognizing catatonia in PD patients, particularly following acute withdrawal from medication. It also emphasizes lorazepam’s diagnostic and therapeutic significance. Catatonia and Parkinson’s disease presentations have comparable motor manifestations, such as stiffness and immobility; nevertheless, the underlying cause of each is distinctive. PD results from dopaminergic degeneration of the substantia nigra pars compacta, impairing bottom-up modulation of basal ganglia circuits (7). In comparison, dysfunction of cortical regions, most significantly the orbitofrontal and premotor cortices, is assumed to lead to catatonia, which interferes with top-down inhibition of subcortical motors (12). In our case, abrupt dopaminergic withdrawal may disrupt an already fragile inhibitory network, triggering catatonia rather than Parkinsonism. Moreover, the patient’s marked improvement following lorazepam administration further supports the diagnosis of catatonia and help differentiates it from extrapyramidal symptoms, which typically do not respond to benzodiazepines (13). Clinical experience has consistently demonstrated such immediate reversal of such symptoms with lorazepam, confirming its diagnostic application in catatonia (14) The patient’s rapid and sustained response to lorazepam, a GABA-A agonist that exerts sedative and inhibitory effects (15), further confirming the diagnosis. This is compatible with established evidence supporting lorazepam’s role in restoring cortical-subcortical inhibitory balance by potentiating GABAergic tone (16). The patient’s acromegaly may have contributed to the patient’s neuropsychiatric vulnerability. While a direct link to catatonia has not yet been established, chronic elevation of IGF-1 changes the microstructure of the cortex and white matter in acromegaly, which may contribute to neuropsychological impairment, affecting mood and cognition (17). One previous case also reported similar structural brain changes in a patient with acromegaly, supporting the possible link between GH/IGF-1 excess and neuropsychiatric symptoms (18). These factors may have lowered her threshold for decompensation in the context of Parkinson’s disease and medication disruption. In addition, the imaging and history of previous midbrain infarcts and cerebral microangiopathy suggest that cerebrovascular disease may have disrupted frontostriatal and thalamocortical circuits. Several case reports have documented the occurrence of catatonia after ischemic damage to the basal ganglia, thalamus, or midbrain, showing how vascular damage can predispose to neuropsychiatric syndromes by disrupting these networks (19–21). These multifactorial contributors support the evolving understanding of catatonia as a disorder of disrupted connectivity within the brain, including the frontal, parietal, and basal ganglia regions. Irrespective of whether these pathologies are due to neurodegeneration, endocrine disturbance, or cerebrovascular injury, they can overlap to produce the typical phenomenology of catatonia (22). In our patient, these overlapping causes which include Parkinson’s disease, acromegaly, and prior vascular injury, may have worked together to trigger catatonia. The early recovery on lorazepam before the start of Parkinson’s treatment and the marked improvement in her overall health may be attributed to the concurrent treatment of acromegaly.

## Conclusion

This case highlights the importance of recognizing catatonia and psychosis as potential complications of Parkinson’s disease, particularly in the context of drug withdrawal and concomitant acromegaly. Lorazepam worked effectively on these psychiatric symptoms and showed its effectiveness in the treatment of these co-occurring complex conditions.

## Data Availability

The original contributions presented in the study are included in the article/supplementary material. Further inquiries can be directed to the corresponding author.
